# Regadenoson myocardial perfusion scintigraphy for the evaluation of coronary artery disease in patients with lung disease: A series of five cases

**DOI:** 10.1007/s12350-019-01956-w

**Published:** 2019-12-03

**Authors:** Eliana Reyes, Stephen Richard Underwood

**Affiliations:** 1 Royal Brompton & Harefield NHS Foundation Trust, London, UK; 2 The PET Imaging Centre, St Thomas’ Hospital, King’s College London, London, UK; 3 National Heart & Lung Institute, Imperial College London, London, UK

**Keywords:** Regadenoson, myocardial perfusion scintigraphy (MPS), coronary artery disease (CAD), lung disease, vasodilators

## Abstract

Coronary artery disease (CAD) is a leading cause of death and morbidity globally. Myocardial perfusion scintigraphy (MPS) is commonly used for the diagnosis of CAD, necessitating hyperaemia achieved either by physical exertion or by pharmacological stress, most commonly through use of a coronary arteriolar dilator. This is challenging in patients with respiratory conditions because exercise may be submaximal and adenosine is contraindicated because of the risk of bronchoconstriction. Regadenoson is the only selective adenosine A_2A_ receptor agonist approved as a vasodilator in MPS. The risk of bronchospasm with regadenoson has been investigated in large, randomised trials; however, patients with the most severe respiratory conditions were not included. In this case series, we present the use of regadenoson MPS in five patients with moderate-to-severe lung conditions, including patients requiring lung volume reduction surgery and lung transplant. In all cases, regadenoson MPS provided valuable information for risk assessment and treatment optimisation. Although dyspnoea occurred in all patients, regadenoson was well tolerated without serious adverse events or bronchospasm; in no case was intervention required to treat dyspnoea.

## Introduction

Coronary artery disease (CAD) is a global health issue and the leading cause of death and morbidity worldwide.^1^ Myocardial perfusion scintigraphy (MPS) is a well-established, non-invasive technique that provides accurate diagnosis and risk stratification for patients with known or suspected CAD.^2^-^4^ Stress MPS uses exercise and/or pharmacological stressors to increase myocardial perfusion and to detect regional reduction in myocardial perfusion reserve—an early consequence of coronary obstruction.^1^,^2^

Dynamic exercise is a widely used stressor for MPS; however, it either cannot be completed or is contraindicated in a number of patients, namely those who are unable to exercise maximally and those with high-risk cardiac and lung conditions that prevent them from achieving an adequate heart rate.^5^,^6^ Two classes of pharmacological stress agents, vasodilators and inotropic agents, are used clinically to augment or replace exercise stress in these patients.^5^ Vasodilators increase myocardial perfusion by stimulating adenosine A_2A_ receptors, leading to coronary arteriolar dilatation.^6^ However, vasodilators such as adenosine and dipyridamole are non-selective adenosine receptor agonists that can cause heart block or bronchospasm by virtue of their action on A_1_, A_2B_ and/or A_3_ receptors.^6^ Bronchospasm is of particular concern in patients with lung disease such as asthma or chronic obstructive pulmonary disease (COPD).^7^

Regadenoson is the only selective adenosine A_2A_ receptor agonist approved for use with MPS.^8^ It is indicated for the induction of pharmacological stress in patients with suspected or known CAD who are unable to achieve an adequate heart rate through exercise alone because of physical or psychological limitations.^6^,^8^ It is administered as a single fixed-dose injection into a peripheral vein.^8^ After injection, maximum hyperaemia persists for at least 19 seconds in 90% of patients and can last up to 10 minutes in individual patients, hence it does not require continuous infusion.^3^,^8^

Two large randomized clinical trials included 2799 patients and showed that regadenoson MPS has comparable efficacy to adenosine MPS for the detection of inducible perfusion abnormality.^9^,^10^ Regadenoson provided almost identical results to adenosine for ischaemic perfusion defect size, left ventricular ejection fraction (LVEF) and ischaemia severity.^11^ Regadenoson had a more favourable side effect profile than adenosine, with a lower summed symptom score of flushing, chest pain and dyspnoea as well as overall favourability by patients.^9^,^10^ The most common symptoms were dyspnoea (29%), headache (27%), flushing (23%), chest pain (19%), electrocardiogram (ECG) ST segment changes (18%), gastrointestinal discomfort (15%) and dizziness (11%).^8^

Several studies have shown that regadenoson is well tolerated in patients with asthma and/or COPD^7^,^12^-^14^ and, uniquely among MPS stress agents, it is approved for use, with caution, in these patients.^8^ In these studies, there were no significant changes in lung function and severe bronchospasm was not reported, although dyspnoea was more frequently reported with regadenoson than with placebo; however, these studies did not include patients with severe lung disease.^7^,^12^-^14^ The following cases illustrate the value of regadenoson MPS for the detection of CAD in patients with moderate or severe lung disease.

## Case Study 1: MPS in a Patient with Idiopathic Pulmonary Fibrosis and Reduced Exercise Capacity

A retired 75-year-old man had not smoked for 40 years. He presented with worsening shortness of breath, limiting him to one flight of stairs and leading to his sleeping in the living room. He required support with activities of daily living, and he was unable to complete a 6-minute walk test because of desaturation. He used continuous oxygen during which his resting oxygen saturation was 89%.

**Figure 1:**
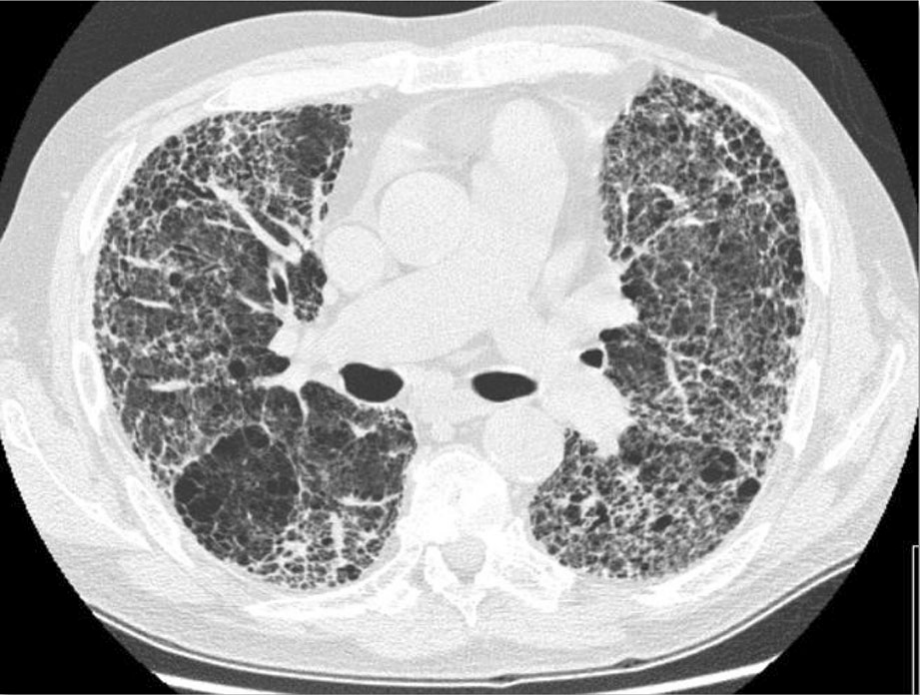
Case 1: CT scan of a retired 75-year-old man, ex-smoker, showing idiopathic pulmonary fibrosis. *CT* computed tomography

**Figure 2:**
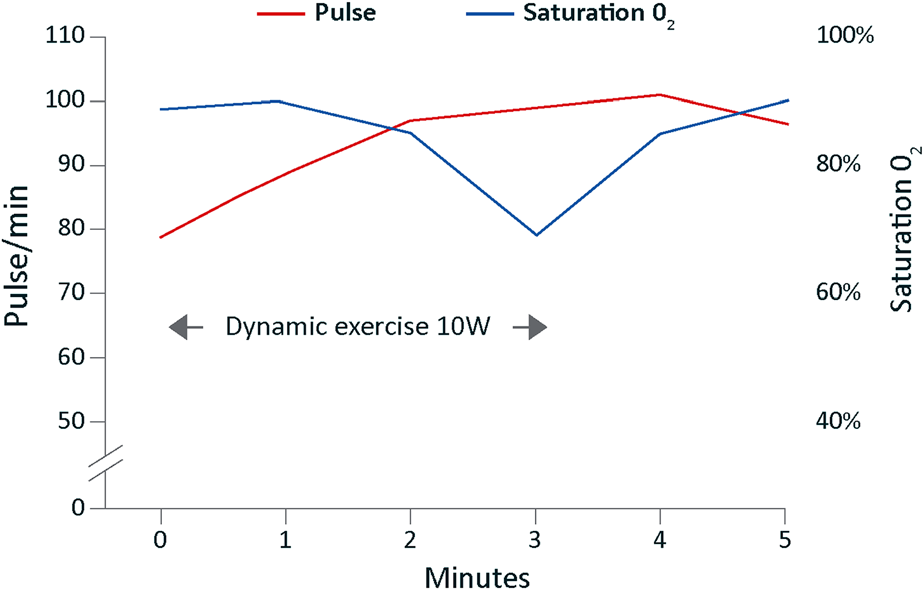
Case 1: oxygen saturation and heart rate of a 75-year-old man, ex-smoker, during regadenoson MPS with dynamic exercise. *MPS* myocardial perfusion scintigraphy

Chest X-ray and X-ray-computed tomography (CT) showed characteristic changes of idiopathic pulmonary fibrosis (Figure 1). Lung function tests showed severe reduction in gas transfer with relatively preserved volumes (Table 1). He also reported exertional chest discomfort and MPS was requested for the investigation of possible CAD. A 1-day stress-redistribution protocol was used with 80 MBq thallium and regadenoson stress combined with bicycle exercise at 10 Watts. His pulse increased from 79 to 101 beats per minute (bpm), blood pressure from 160/90 to 185/95 mmHg, and oxygen saturation on 4 L·min^−1^ oxygen fell from 99 to 79%, resulting in marked breathlessness, at which point exercise was terminated and oxygen saturation rapidly returned to 100% (Figure 2).

**Table 1:** Case 1: lung function tests of retired 75-year-old man, ex-smoker

Lung function	Measurement (% predicted)
FEV_1_	2.58 L (92%)
FVC	3.05 L (82%)
TL_CO_	2.22 (27%)
K_CO_	0.51 (41%)

*FEV*_*1*_ forced expiratory volume in 1 second, *FVC* forced vital capacity, *K*_*CO*_ transfer coefficient, *TL*_*CO*_ carbon monoxide transfer factor

**Figure 3:**
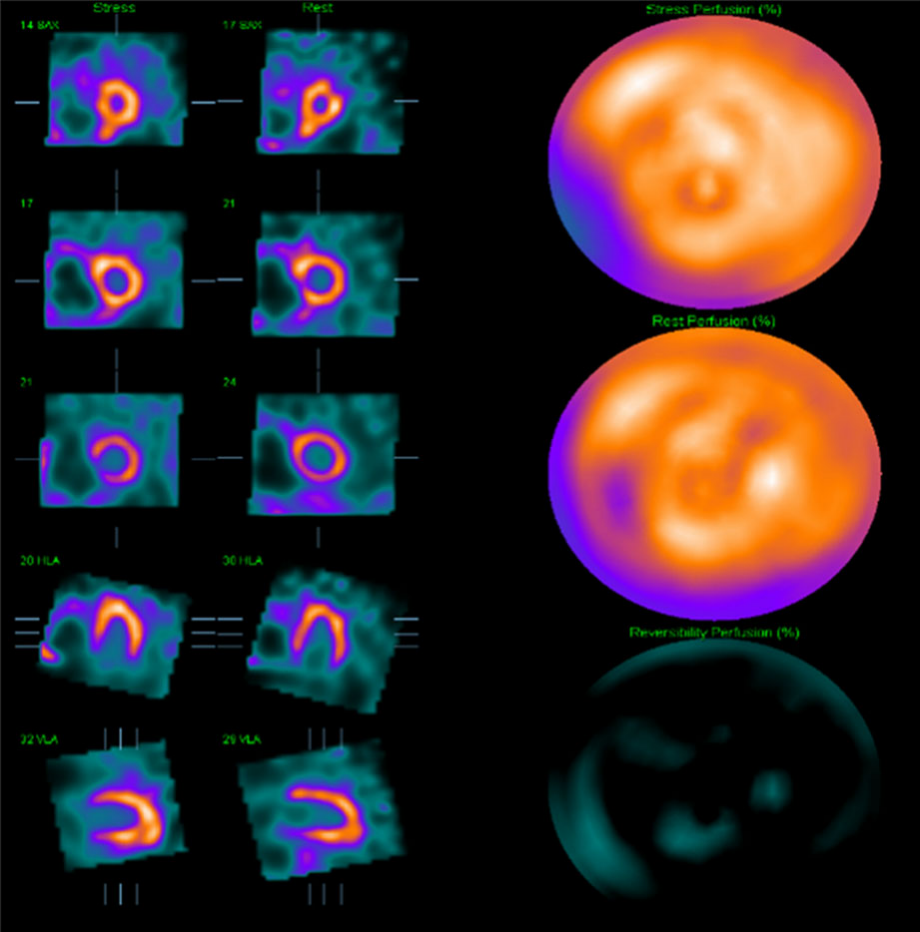
Case 1: Thallium-201 MPS of a 75-year-old man with idiopathic pulmonary fibrosis showing no inducible perfusion abnormality. MPS, myocardial perfusion scintigraphy

The MPS images were normal with no inducible perfusion abnormality and normal resting left ventricular function with an ejection fraction of 55% (Figure 3). However, there was a dilated and hypertrophied right ventricle with severely impaired function and a flattened septum, compatible with pulmonary hypertension (Figure 3). It was concluded that there was no obstructive coronary disease and the patient was accepted for lung transplantation. Unfortunately, the patient died of respiratory failure 3 months later before a transplant became available.

## Case Study 2: Stress MPS in a Patient with Chronic Respiratory Disease Complicated by Recent Type II Respiratory Failure

A 78-year-old man had idiopathic pulmonary fibrosis and type II respiratory failure exacerbated by recent influenza B infection and managed by non-invasive ventilation and long-term oxygen therapy. He also suffered from sleep apnoea and had previous fundoplication for hiatus hernia. He presented with worsening breathlessness on exertion and episodic chest pain at rest, which raised the suspicion of CAD.

Stress was performed with regadenoson and bicycle ergometer exercise at 0 Watts. His heart rate increased from 109 to 123 bpm, and blood pressure changed from 135/85 to 140/70 mmHg, whereas oxygen saturation on room air increased from 84 to 94% at 4 L·min^−1^ of oxygen and remained unchanged throughout the test. He experienced dyspnoea but no other significant symptoms.

**Figure 4:**
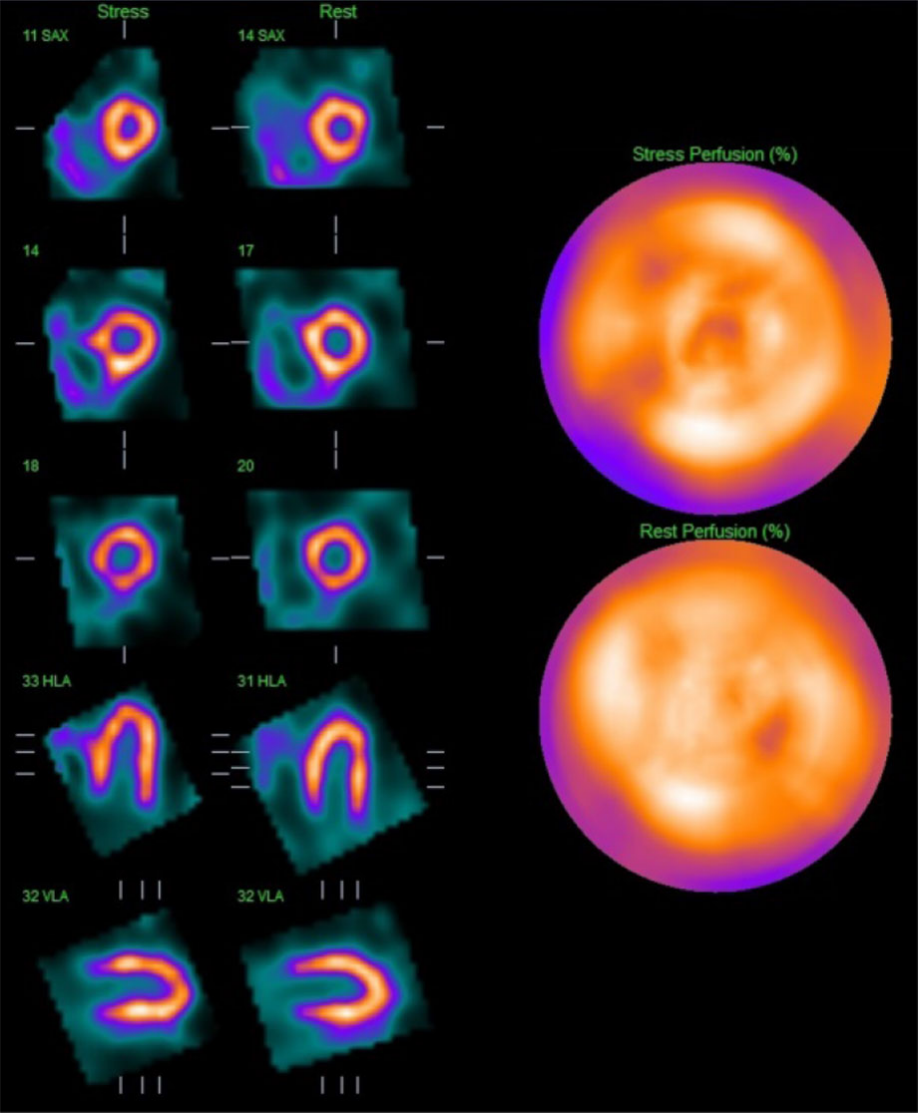
Case 2: Thallium-201 MPS of a 78-year-old man with a history of chronic respiratory disease complicated by recent type II respiratory failure showing no inducible perfusion abnormality. There is no significant inducible perfusion abnormality but the right ventricle is enlarged. *MPS* myocardial perfusion scintigraphy

The MPS images showed no inducible perfusion abnormality and hence no evidence of flow-limiting coronary obstruction underlying his symptoms (Figure 4). Resting left ventricular function was normal (LVEF in excess of 75%) but the right ventricle was dilated and hypertrophied with preserved global function. Further investigation indicated pulmonary hypertension with an estimated pulmonary artery systolic pressure of 53 mmHg. Coronary angiography was not deemed necessary and medical management of his lung condition was continued.

## Case Study 3: Stress MPS in a Patient with Interstitial Lung Disease (ILD) and Pulmonary Hypertension

A 75-year-old woman with a history of ILD secondary to undifferentiated connective tissue disease and pulmonary hypertension presented with palpitation, breathlessness and recent impairment in left ventricular function (LVEF had decreased from 50 to 35% on echocardiography). She also had diabetes and atrial fibrillation, which was managed with digoxin and warfarin. Her exercise tolerance and mobility were limited, and she was supported with long-term oxygen therapy at 2.5 L·min^−1^. Stress MPS was requested because of suspected CAD.

Stress was performed with regadenoson combined with bicycle ergometer exercise at 0 Watts. The patient’s heart rate rose from 71 to 90 bpm and blood pressure from 140/90 to 185/100 mmHg. She experienced mild dyspnoea but no other significant symptoms.

**Figure 5:**
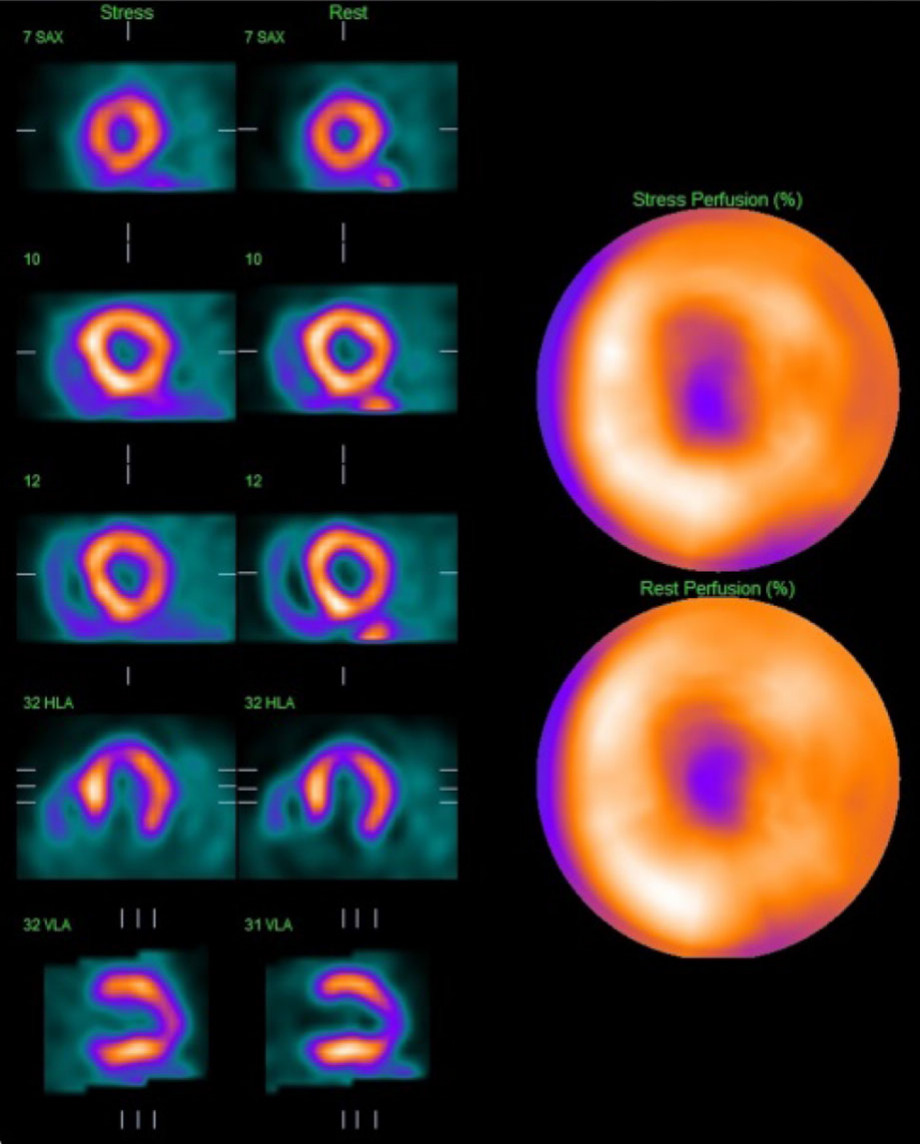
Case 3: Tc-99m tetrofosmin MPS of a 75-year-old woman with ILD and pulmonary hypertension showing mild septal flattening, a partially reversible apical anteroseptal defect and possible (minor) defect in the basal lateral wall. ILD, interstitial lung disease; MPS, myocardial perfusion scintigraphy; Tc, technetium

The MPS images showed an apical anteroseptal defect and a possible defect in the basal lateral wall, both showing little reversibility (Figure 5). These were thought to be the result of attenuation artefact; however, minor true perfusion abnormalities could not be excluded with certainty. Given the extent of the defects, it was concluded that the scintigraphic appearances would support medical therapy in the first instance. Resting left ventricular function was normal (LVEF, 55%) with mild septal flattening. Following stress MPS, medical therapy was optimised, and a small dose of beta-blocker was added. Coronary angiography was not performed. The patient subsequently presented with acute fluid overload, most likely related to underlying atrial fibrillation. She responded satisfactorily to medical management. At 12 months following stress MPS, she was clinically stable.

## Case Study 4: MPS in a Patient with Late-Stage COPD Undergoing Risk Assessment for Lung Transplantation

A 60-year-old woman had a 40 pack-year smoking history and severe COPD (Global Initiative for Chronic Obstructive Lung Disease [GOLD] stage IV). She was also known to have pulmonary fibrosis and severe pulmonary hypertension and was being assessed for lung transplantation. She was breathless at rest and was supported by continuous oxygen therapy. MPS was requested to exclude CAD and regadenoson stress was selected.

**Figure 6:**
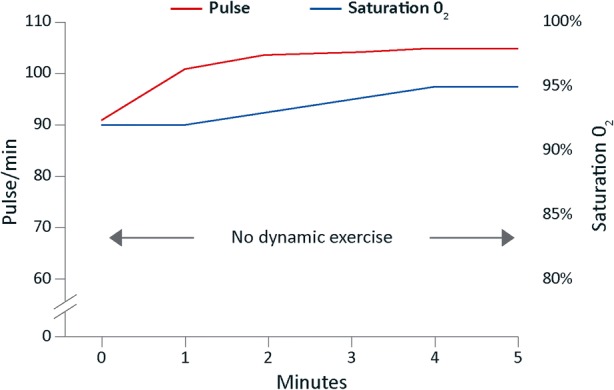
Case 4: oxygen saturation and heart rate of a 60-year-old woman during MPS investigation with regadenoson without dynamic exercise. MPS, myocardial perfusion scintigraphy

Regadenoson 400 µg was given intravenously; after 1 minute she became both tachypnoeic and dyspnoeic and was using her accessory muscles of respiration. Pulses increased from 91 to 104 bpm and blood pressure fell from 140/70 to 135/50 mmHg; however, oxygen saturation increased from 92 to 95% indicating that the dyspnoea was not secondary to hypoxia (Figure 6). The dyspnoea resolved 2 minutes later.

**Figure 7:**
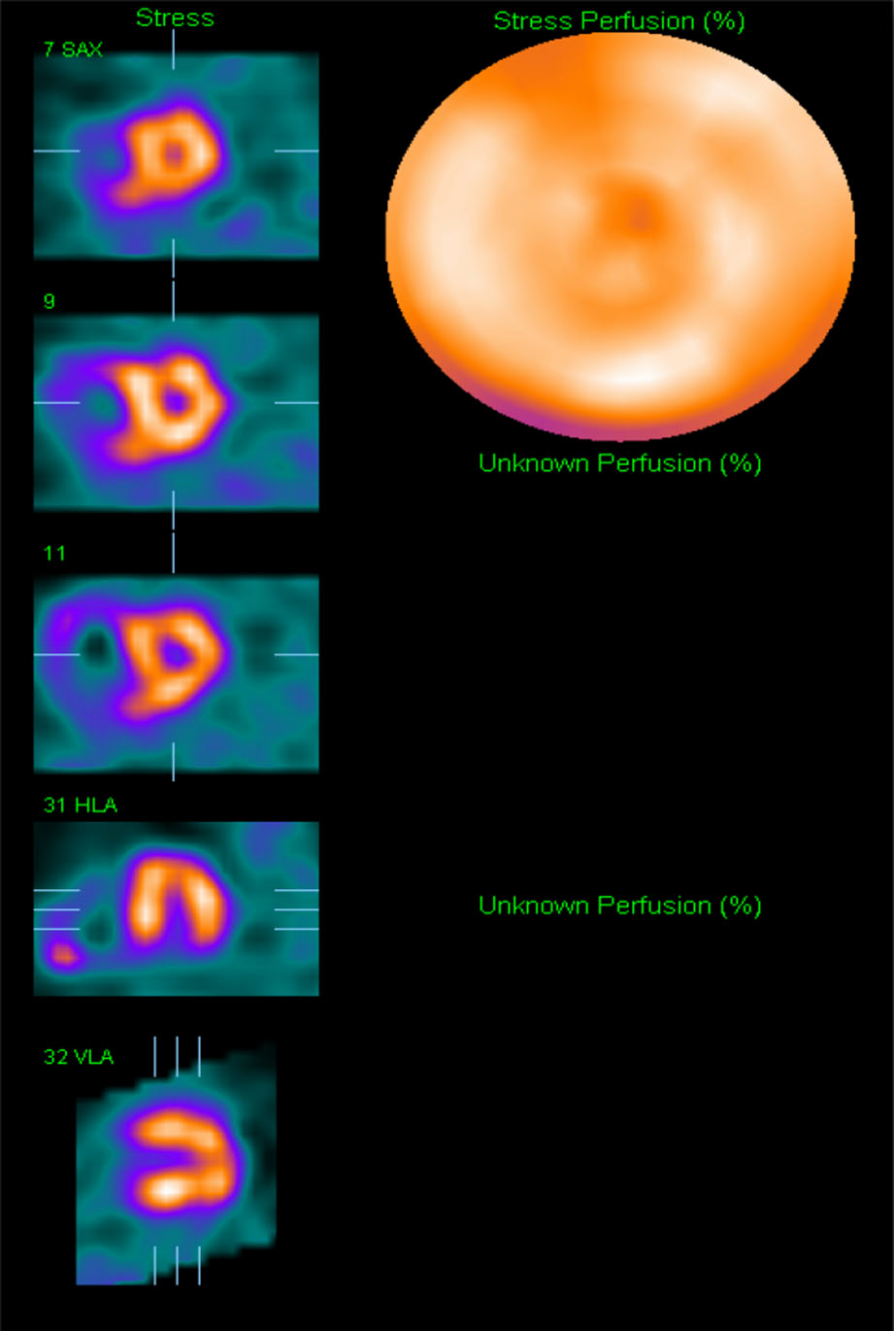
Case 4: Tc-99m tetrofosmin MPS of a 60-year-old woman with severe COPD and pulmonary fibrosis showing homogeneous stress perfusion throughout the left ventricular myocardium and signs of pulmonary hypertension. Resting images were not acquired. COPD, chronic obstructive pulmonary disease; MPS, myocardial perfusion scintigraphy; Tc, technetium

The MPS images showed normal stress perfusion throughout the left ventricle and a resting study was not performed. There was septal flattening and right ventricular hypertrophy consistent with pulmonary hypertension (Figure 7). The patient was accepted for lung transplantation but unfortunately died of respiratory failure before a transplant became available.

## Case Study 5: Stress MPS for Pre-operative Risk Assessment Before Lung Volume Reduction Surgery (LVRS)

The patient was an active 72-year-old man with a history of CAD and severe emphysema (GOLD stage III). Cardiovascular risk factors included a 33 pack-year exposure to smoking, hypertension and hyperlipidaemia. Several years previously, he had an acute myocardial infarction and underwent coronary artery bypass graft surgery, following which he had no significant cardiac symptoms. He presented with exertional breathlessness. Lung function testing showed FEV1 (forced expiratory volume in one second) 44% of predicted, while a chest CT scan demonstrated bullous destruction of the lungs. Following assessment, LVRS was considered and stress MPS was requested to assess pre-operative risk.

Regadenoson was combined with exercise on a bicycle ergometer at 0 Watts. Heart rate increased from 65 to 90 bpm, and blood pressure changed from 160/70 to 135/85 mmHg; oxygen saturation changed from 96 to 97% at peak stress but fell to 89% by the end of the test. The patient experienced mild flushing and dyspnoea but no other significant symptoms. There were no ECG changes.

**Figure 8:**
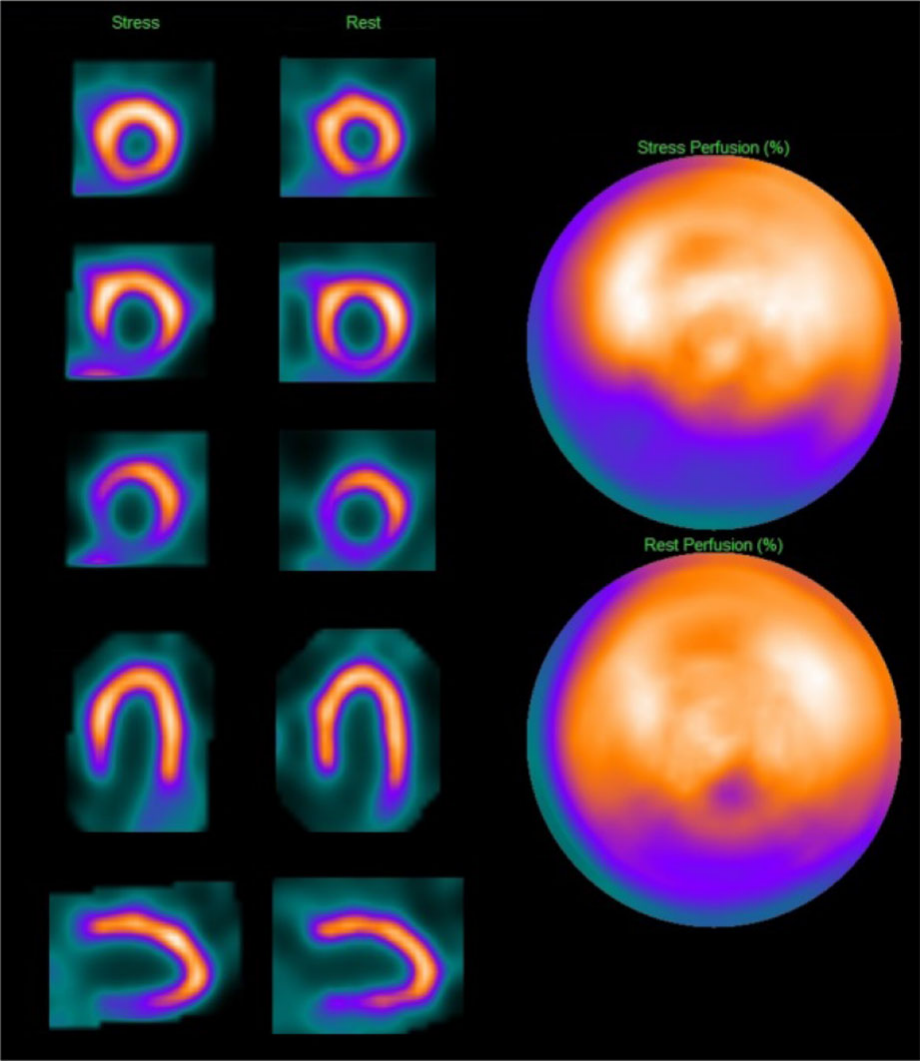
Case 5: Tc-99m tetrofosmin MPS of a 72-year-old man with previous coronary artery bypass graft and severe emphysema showing partial-thickness inferior infarction with mild-to-moderate superimposed and peri-infarct ischaemia. MPS, myocardial perfusion scintigraphy; Tc, technetium

The MPS images showed a partially reversible defect in the inferior wall extending into the inferoseptal region (Figure 8). ECG-gating of the resting tomograms showed reduced thickening of the inferior wall and basal inferoseptal region with normal global left ventricular function (LVEF, 60%). The scintigraphic appearances were in keeping with partial-thickness inferior infarction with mild-to-moderate superimposed and peri-infarct ischaemia. The ischaemic burden was estimated at 9% of total left ventricular myocardium. The risk of peri-operative ischaemic events was considered to be intermediate.

The case was discussed by a multidisciplinary team, and consensus reached in favour of continuing medical therapy. A 12-month clinical review was scheduled to re-assess the indication for LVRS.

## Discussion

This series of cases confirms the suitability of regadenoson stress for MPS without significant adverse events in patients with severe lung disease and with pulmonary hypertension. The patients described had severe lung disease such as COPD and idiopathic pulmonary fibrosis, including patients with pulmonary hypertension, respiratory failure, and with disease advanced enough to consider LVRS/transplantation. MPS is commonly required in such patients either to assess possible underlying CAD or for peri-operative risk assessment.

Notably, although dyspnoea occurred in all patients, it was well tolerated without serious adverse events or bronchospasm. In case 1, the fall in oxygen saturation was likely related to the concomitant exercise because there was rapid recovery upon cessation. In case 4, dyspnoea was associated with an increase in saturation suggesting that the dyspnoea and tachypnoea were centrally mediated. In these, as in the other cases, dyspnoea was not related to bronchospasm or deterioration of lung function. The exact mechanism by which vasodilator stress agents induce dyspnoea remains uncertain. The results from a previous study suggest that dyspnoea may occur as a result of stimulation of adenosine A_1_ and A_2A_ receptors located in the pulmonary vagal afferent C-fibres.^15^

This series also illustrates the need to monitor oxygen saturation levels to ensure that modifications to the stress test are appropriate. No medical intervention was required because of regadenoson-induced symptoms. The ability to conduct regadenoson MPS provided valuable information for risk assessment and treatment optimisation in these patients.

## Conclusions

These cases illustrate the safety and tolerability of regadenoson in patients with moderate-to-severe lung-related comorbidities and describe how the results of these tests may be valuable for patient management.
